# Silencing LY6K Suppresses CD44^+^ EpCAM^+^ HCT116 Human Colon Cancer Stem Cells Growth: Insights from In Vitro and In Vivo Evidence

**DOI:** 10.3390/cimb46120840

**Published:** 2024-12-12

**Authors:** Changhao Fu, Kuiqiao Chen, Jinyue Duan, Kun Liu, Miaomiao Li, Yuanyuan Chen, Zhongyi Cong, Yi Wang

**Affiliations:** 1 Department of Regenerative Medicine, School of Pharmaceutical Sciences, Jilin University, Changchun 130021, Chinachenkq16@mails.jlu.edu.cn (K.C.); duanjy18@mails.jlu.edu.cn (J.D.); kunliu20@mails.jlu.edu.cn (K.L.); limm21@mails.jlu.edu.cn (M.L.); muyao1221@foxmail.com (Y.C.); congzhy@jlu.edu.cn (Z.C.); 2 School of Medicine, Stanford University, Palo Alto, CA 94304, USA

**Keywords:** colon cancer, RNA interference, tumor growth, survival, LY6K

## Abstract

Lymphocyte antigen 6 complex, locus K (LY6K) is a putative oncogene in various human cancers, including colorectal cancer, where elevated expression is associated with poor prognosis. This study investigates the antitumor effects of LY6K in colon cancer stem cells (CCSCs) both in vitro and in vivo. EpCAM and CD44 surface markers were used to isolate CCSCs from HCT116 cells, and the expression of LY6K in CCSCs was analyzed by real-time PCR. RNA interference was used to silence LY6K to evaluate its potential role of LY6K on the proliferation, migration, invasion, and cell cycle of CCSCs. Functional assays, including MTS assays, flow cytometric analyses, Transwell migration and invasion assays, and a xenograft model, were used for analysis. The results revealed that LY6K was highly expressed in CCSCs. siRNA-mediated LY6K-silencing in CCSCs inhibited cell proliferation by inducing G1 phase cell cycle arrest and suppressed migration and invasion. In vivo, LY6K silencing effectively reduced tumor growth and extended survival in a mouse model. These findings suggest LY6K as a promising therapeutic target for eradicating CCSCs in colorectal cancer treatment.

## 1. Introduction

Colorectal cancer is the third leading cause of cancer deaths in the United States, characterized by high mortality rates and poor five-year survival outcomes [1]. One major reason for therapy failure is the presence of residual cell populations enriched in cancer stem cells (CSC) within the tumor, which are inherently resistant to chemo- and radiotherapy [2]. CSCs are a subgroup of cancer cells capable of self-renewal and differentiation into heterogeneous cancer cell types [3]. They exist in various solid tumors and are responsible for intertumoral heterogeneity, which contributes to tumor initiation, progression, metastasis, and relapse [4,5]. Identifying molecular targets to eliminate CSCs is critical for improving colorectal cancer treatment.

The lymphocyte antigen-6 (Ly6)/urokinase-type plasminogen activator receptor (uPAR) superfamily was initially described in mice, with the subsequent identification of conserved human homologs across different species, which exhibit various expression patterns and functions [6,7]. The human Ly6/uPAR gene family is mapped to chromosomes 8, 19, 6, and 11, and currently include 35 members, which are classified into two subfamilies based on the absence or presence of the glycosylphosphatidylinositol (GPI)-anchor domain. Human Ly6/uPAR proteins participate in critical biological processes, including cancer growth, immune regulation, and inflammation [6,8,9,10]. LY6E, LY6H, LY6K, and prostate stem cell antigen (PSCA) are Ly6 family members that are mapped to chromosome 8q24.3. Studies have demonstrated that they are expressed at high levels in multiple human cancers, are involved in tumor progression, and promote immune escape, which is correlated with poor survival outcomes [9,10]. Among the Ly6/uPAR members, LY6K (also named cancer-testis antigen) has been identified as a putative oncogene with elevated expression in several cancers, such as breast, head-and-neck, bladder, lung, and eraesophageal cancers. The overexpression of LY6K often correlates with poor outcomes and poor overall survival [11,12]. LY6K also promotes drug resistance and epithelial-to-mesenchymal transition that contribute to tumor growth [13]. Additionally, LY6K has been shown to promote invasion and metastasis by upregulating matrix metalloproteinase proteins (MMP-2 and MMP-9) in breast cancer [14]. The causal mechanisms underlying LY6K’s oncogenic potential remain to be elucidated, but studies implicate its involvement in crucial signaling pathways, including TGF-β signaling, Raf-1/MEK/ERK signaling, and immune regulation [11,12,13,14]. In colorectal cancer, analyses using Oncomine and the Georgetown Database of Cancer have revealed elevated LY6K mRNA levels in tumor tissues compared to adjacent normal tissues, correlating with poor prognosis [15]. However, the functional role of LY6K in colorectal cancer remains largely unexplored. 

In our previous studies, proteins from the Ly6 family, LY6D, have been identified to be upregulated in colon CSCs, where they are implicated in tumor progression and CSC maintenance, while research on LY6K in colon CSCs is limited [16]. Since colon CSCs are often linked to resistance to chemotherapy and recurrence, exploring the role of LY6K in these cells could be valuable for developing targeted therapies that may improve treatment outcomes. This study investigates the expression and function of LY6K in colon cancer stem cells (CCSCs), both in vitro and in vivo.

## 2. Materials and Methods

### 2.1. Animals and Cells Lines

BALB/c nude mice (female, 6–8 weeks old) from Beijing HFK Bioscience Co., Ltd. (Beijing, China) were housed in the Laboratory Animal Center at Jilin University (Changchun, China). The human colon cancer HCT116 cell line from The Cell Bank of the Chinese Academy of Sciences (Shanghai, China) was cultured in RPMI-1640 media (Gibco; Thermo Fisher Scientific, Waltham, MA, USA) and supplemented with 10% fetal bovine serum (FBS, Gibco) at 37 °C in a humidified atmosphere containing 5% CO_2_. 

### 2.2. Magnetic-Activated Cell Sorting (MACS)

EpCAM^+^ CD44^+^ HCT116 cells (namely CCSCs) were isolated from HCT116 cells using the CELLection Biotin Binder kit (Invitrogen, Carlsbad, CA, USA; Thermo Fisher Scientific, Waltham, MA, USA). According to the manufacturer’s instructions, 4 × 10^7^ HCT116 cells were incubated sequentially with biotin-conjugated EpCAM antibodies and biotin-conjugated CD44 antibodies at 4 °C for 20 min. Magnetic separation was performed to obtain CCSCs. The sorted CCSCs were cultured in DMEM/F12 (Gibco) that was supplemented with 20 ng/mL of both epidermal growth factor (EGF) and basic fibroblast growth factor (bFGF; both from PeproTech, Rocky Hills, Cranbury, NJ, USA) and 2% B27 (Gibco) at 37 °C and 5% CO_2_.

### 2.3. Sphere Formation Assay

Sphere formation assays were performed as previously described [17]. Briefly, CCSCs were plated on uncoated six-well plates at a density of 1 × 10^4^/mL in a medium containing DMEM/F12 supplemented with 2% B27 and 20 ng/mL of both EGF and bFGF. Tumorspheres were visualized for 7 days and images were obtained by an inverted microscope (Olympus Corporation, Tokyo, Japan). 

### 2.4. Serum-Induced Differentiation of Tumorspheres

Tumorspheres were resuspended in a RPMI-1640 medium containing 10% FBS and cultured for 7 days [18]. Images of CCSC spheres were captured using an inverted microscope (Olympus Corporation).

### 2.5. RNA Extraction and Quantitation 

The total RNA was extracted using a TRIzol reagent (Invitrogen) according to the manufacturer’s protocol. cDNA was synthesized by reverse transcription using the PrimeScript RT Reagent Kit (Takara Bio, Beijing, China). The target gene expression levels were quantified via qPCR analysis using SYBR Premix Ex Taq (Takara Bio) on an 7500 Real-Time PCR System (Applied Biosystems; Thermo Fisher Scientific). The relative expression levels of each gene were normalized to GAPDH levels using the 2^−∆∆Cq^ method [19,20]. Primer sequences are listed in Table 1. 

### 2.6. Transfection of Small Interfering RNA (siRNA)

siRNA targeting LY6K (siLY6K; 5′-GAACCCAAGGAGGTGCAAA-3′, 50 nM) and negative-control siRNAs (siNC, 50 nM) were obtained from RiboBio (RiboBio, Guangzhou, China). Transfections to CCSCs were performed using the RiboFECT^TM^CP transfection reagent (RiboBio) according to the manufacturer’s protocol. Transfected cells were harvested after 48 h to measure the silencing efficiency of siLY6K.

### 2.7. Western Blot Analysis

Cells were harvested and lysed in a RIPA lysis buffer containing protease inhibitors (Dingguo Changsheng Biotechnology Co., Ltd., Beijing, China). The total protein was extracted from the cells, and the protein concentrations were determined using the Bicinchoninic Acid Assay Kit (Beyotime, Nanjing, Jiangsu, China). The proteins were separated via SDS-PAGE, transferred to polyvinylidene difluoride membrane, and blocked with 5% skimmed milk in a Tris-buffered saline for 1 h at room temperature. The membranes were incubated overnight at 4 °C with primary antibodies [LY6K (1:750, #PIPA572689, Invitrogen), MMP-2 (3:1000, #BM0569, Boster, Pleasanton, CA, USA), MMP-9 (1:300, #BM4089, Boster), tissue inhibitor of MMPs-1 (TIMP-1) (3:1000, #BM4980, Boster), cyclin-dependent kinase (CDK) 4 (3:1000, #BM4672, Boster), cyclin D1 (3:1000, #bs-0623R, Bioss), CDK2 (3:1000, #bs-0757R, Bioss), cyclin E1 (3:1000, #bs-0573R, Bioss), caspase-3 and cleaved caspase-3 (1:1000, #14220, CST), β-actin (1:1500, #AF0003, Beyotime), and β-tubulin (1:1000, #AC008, Beyotime)], followed by incubation with secondary antibodies [horseradish peroxidase-conjugated goat anti-rabbit (1:2000, #A0208, Beyotime) and anti-mouse (1:2000, #A0216, Beyotime)] for 1 h at room temperature. The protein bands were visualized using super enhanced chemiluminescence detection reagent (Yeasen Biotechnology, Shanghai, China) by X-ray film and analyzed using Scion Image version 4.0.2 (Scion Corporation, Frederick, MD, USA). Protein expression levels were normalized to β-actin or β-tubulin. 

### 2.8. Cell Migration and Invasion Assays

Transwell assays were performed to measure whether silencing of LY6K inhibits the migration and invasion of CCSCs according to our previous study [21]. Following 48 h of transfection with siLY6K, a total of 2 × 10^5^ CCSCs were placed on the upper chamber of a Transwell insert, while RPMI-1640 with 10% FBS was added in the bottom of the lower chamber. After 48 h of incubation, the CCSCs that adhered to the lower membrane surface were fixed with 4% paraformaldehyde for 10 min and stained with 0.1% (*w*/*v*) crystal violet at room temperature for 20 min. siNC-transfected and non-transfected CCSCs (mock) served as the negative controls. Five randomly selected fields were imaged using an inverted phase-contrast microscope (Olympus Corporation), and the cells were counted.

### 2.9. Cell Proliferation Assay

The effects of LY6K on cell proliferation were evaluated by MTS assay using CellTiter 96 AQueous One Solution Cell Proliferation Assay kit (MTS; Promega Corporation, Madison, WI, USA). According to the manufacturer’s instructions, CCSCs transfected with siLY6K were seeded at a density of 1 × 10^4^ cells/100 μL in each well of a 96-well plate. siNC-transfected and non-transfected CCSCs (mock) were used as the negative controls. Absorbance at 490 nm was measured at 0, 24, 48, and 72 h using a microplate reader (Multiskan GO Microplate Spectrophotometer, Thermo Fisher Scientific). 

### 2.10. Flow Cytometry Analysis of Cell Cycle

To investigate the effects of LY6K on cell cycle, flow cytometric analysis of cell cycle was performed using THE cell-cycle detection Kit (KeyGEN BioTECH, Co., Ltd., Nanjing, China) according to the manufacturer’s instructions. Briefly, 2 × 10^5^ siLY6K-transfected CCSCs were fixed in ice-cold 70% ethanol at 4 °C overnight. Following three washes with PBS, cells were incubated with ribonuclease A and PI at 37 °C in the dark for 30 min. siNC-transfected and non-transfected CCSCs (mock) served as the negative controls. The distribution of the cells in each phase was analyzed by a flow cytometer (FC500, Beckman Coulter, Miami, FL, USA) using CellQuest Pro software (version 6.0, BD Biosciences, San Jose, CA, USA). 

### 2.11. Annexin V-FITC/PI Apoptosis Assay

To investigate if LY6K plays a role in apoptosis, flow cytometric detection of apoptosis was assessed using the Annexin V-FITC/PI Apoptosis Detection kit (KeyGEN BioTECH) according to the manufacture’s protocol. Briefly, 1 × 10^5^ of siLY6K-transfected CCSCs were resuspended in 500 μL Annexin V-binding buffer and sequentially stained with Annexin V-FITC (5 μL) and PI (5 μL) solution in the dark for 15 min at room temperature. siNC-transfected and non-transfected CCSCs were used as the negative control. Cells were analyzed via flow cytometry (FC500, Beckman Coulter, Miami, FL, USA) using CellQuest Pro software (version 6.0, BD Biosciences).

### 2.12. In Vivo Tumor Xenograft Assay

A colon cancer model was established by subcutaneous injection of 1 × 10^6^ CCSCs in the left flank of each BALB/c nude mouse. Once the tumor reached an average size of 50–120 mm^3^, tumor-bearing mice were randomly divided into three groups (n = 5 mice per group): siLY6K, siNC, and mock groups. Mice were treated with intratumoral injections of siLY6K or siNC (5 nmol/10 μL saline) twice a week for three weeks, while the mock group received 10 μL saline. Tumor volumes (V) were measured every three days and estimated by using the formula: V = 0.5 × Length × Width^2^. Mice were sacrificed on Day 31, and the tumor weights were measured. For survival analysis, mice (n = 5 mice per group) were monitored daily for 50 days.

### 2.13. Statistical Analysis

Data are expressed as mean ± standard error (SE). Student’s *t*-test or one-way analysis of variance followed by Tukey–Kramer multiple comparisons tests were used to compare the corresponding data. Kaplan–Meier method and log-rank (Mantel–Cox) tests were used for survival analysis. GraphPad Prism (version 8.0, GraphPad Software, San Diego, CA, USA) was employed for statistical analysis, and a *p*-value < 0.05 was considered statistically significant.

## 3. Results

### 3.1. Cell Identification

**Figure 1:**
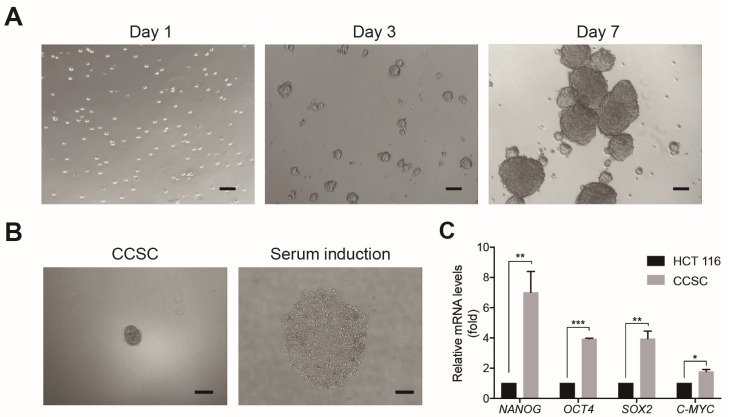
Characterization of CCSCs. (**A**) Culture of CCSCs in a serum-free DMEM/F12 medium for 7 days. Scale bar, 100 μm. n = 3. (**B**) Serum-induced differentiation of the CCSC sphere into epithelioid cells. Scale bar, 100 μm. n = 3. (**C**) RT-qPCR analyses showing relative mRNA levels of target genes in CCSCs compared with HCT116 cells. Values are the mean ± SE (n = 3). * *p <* 0.05, ** *p <* 0.01, or *** *p <* 0.001 indicate significant differences from the HCT116 group as assessed by Student’s *t*-test. CCSC, colon cancer stem cells.

CCSCs were successfully obtained using MACS with CD44 and EpCAM, the most commonly used surface markers for identifying colorectal CSCs [22]. As shown in Figure 1, the identity of CSCs was confirmed by growing CCSCs in a serum-free condition, which gradually formed tumorspheres after cultivation for 7 days. Upon the addition of 10% FBS, the tumorspheres differentiated into adherent confluent cells. qPCR analysis confirmed that stem cell markers NANOG, OCT4, SOX2, and C-MYC were highly expressed in CCSCs compared with HCT116 cells. These results validate that the sorted EpCAM^+^ CD44^+^ HCT116 cells were CCSCs.

### 3.2. High Expression and siRNA-Mediated Silencing of LY6K in CCSCs

**Figure 2:**
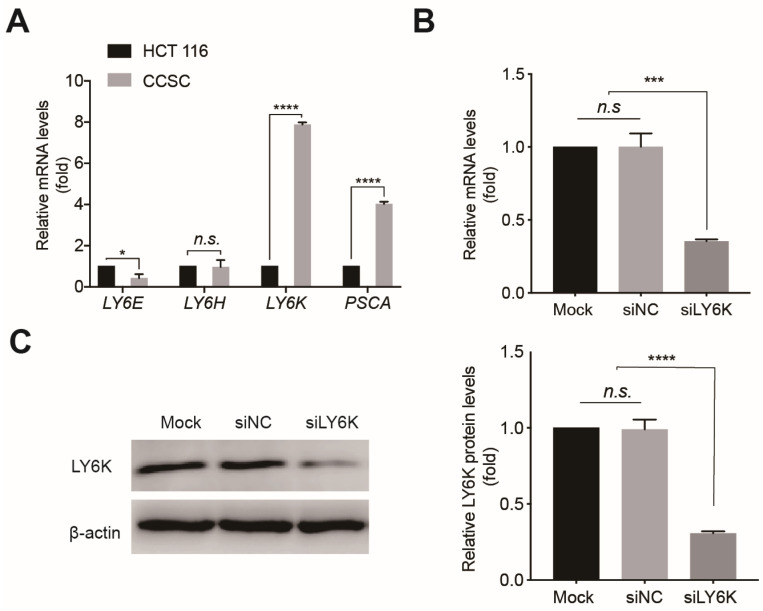
Expression and knockdown of LY6K in CCSCs. (**A**) RT-qPCR analyses showing LY6K mRNA levels in CCSCs and HCT116 cells. Values are the mean ± SE (n = 3). * *p <* 0.05 and **** *p <* 0.0001 indicate significant differences from the HCT116 group as assessed by Student’s *t*-test. RT-qPCR (**B**) and Western blot (**C**) analyses of LY6K knockdown. Values are the mean ± SE (n = 3). *** *p <* 0.001 or **** *p <* 0.0001 indicates significant differences from the Mock and siNC groups as assessed by one-way ANOVA with Tukey–Kramer multiple comparisons tests. LY6K, lymphocyte antigen 6, locus K; CCSC, colon cancer stem cells; n.s., not significant.

qPCR analysis revealed that LY6K and PSCA were upregulated in CCSCs compared to HCT116 cells, while LY6E was downregulated, and no significant differences were observed in LY6H expression (Figure 2A). siRNAs-mediated silencing of LY6K in CCSCs significantly reduced LY6K mRNA (Figure 2B) and protein (Figure 2C) levels, as determined by qPCR and Western blotting analyses, respectively, compared to negative controls. 

### 3.3. LY6K-Silencing Inhibits the Migration and Invasion of CCSCs

**Figure 3:**
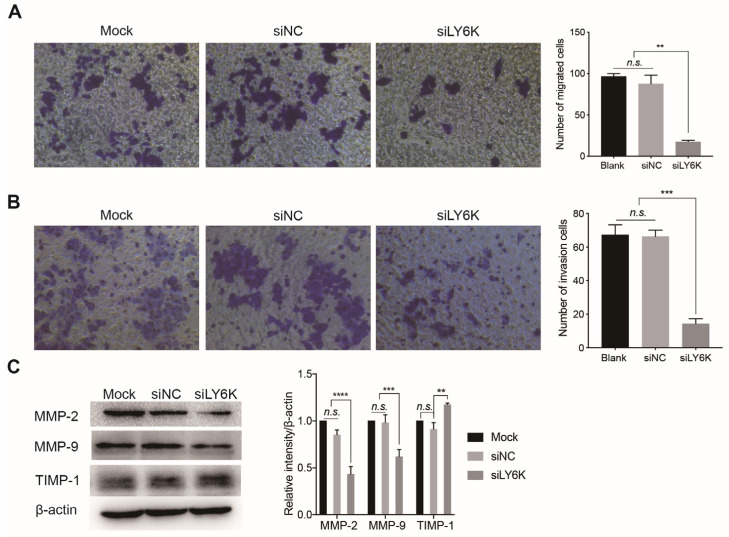
Knockdown of LY6K inhibits migration and invasion of CCSCs in vitro. Optical micrographs and the number of migrated (**A**) and invaded (**B**) CCSCs following siLY6K or siNC transfection for 48 h according to Transwell migration and invasion assay. n = 3. (**C**) Western blot analyses showing the protein levels of MMP-2, MMP-9, and TIMP-1 in CCSCs after treatment with siLY6K or siNC for 48 h. Values are the mean ± SE (n = 3). ** *p* < 0.01, *** *p* < 0.001, or **** *p* < 0.0001 indicates significant differences from the Mock and siNC group as assessed by one-way ANOVA with Tukey–Kramer multiple comparisons tests. LY6K, lymphocyte antigen 6, locus K; CCSC, colon cancer stem cells; MMP, matrix metalloproteinase; TIMP-1, tissue inhibitor of MMP-1; n.s., not significant.

Transwell migration and invasion assays showed that siRNA-mediated silencing of LY6K inhibited the migratory (Figure 3A) and invasive (Figure 3B) abilities of CCSCs compared to negative controls. Western blot analysis demonstrated that MMP-2 and MMP-9 protein levels were downregulated, while TIMP-1 was upregulated in CCSCs transfected with siLY6K compared with controls (Figure 3C). 

### 3.4. LY6K-Silencing Inhibits Cell Proliferation by Inducing G1 Cell-Cycle Arrest in CCSCs 

**Figure 4:**
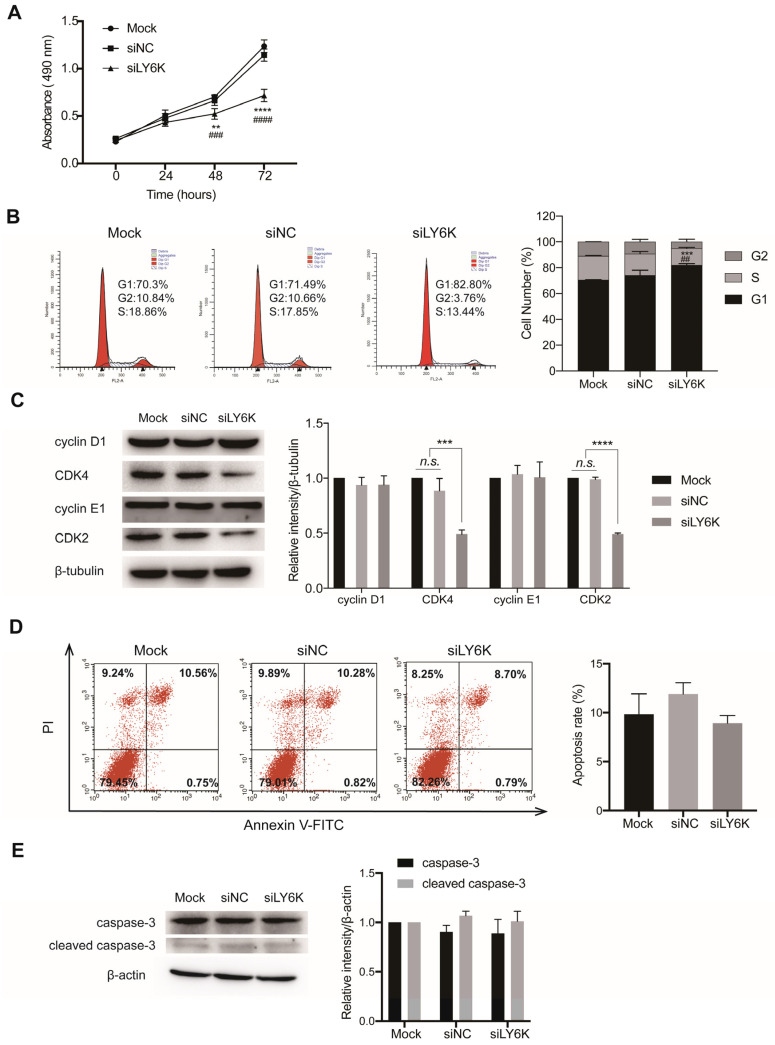
LY6K knockdown inhibits cell proliferation by causing cell-cycle arrest in the G1-phase. (**A**) MTS assays showing the proliferation potential of CCSCs after siLY6K transfection. Values are the mean ± SE (n = 3). ** *p* < 0.01 or **** *p* < 0.0001 and ^###^ *p* < 0.001 or ^####^ *p* < 0.0001 indicate significant differences from the Mock and siNC groups, respectively, as assessed by one-way ANOVA with Tukey–Kramer multiple comparisons tests. (**B**) Flow cytometric analyses showing the cell-cycle progression in CCSCs and number of CCSCs in each cell-cycle phase. Values are the mean ± SE (n = 3). ^##^ *p* < 0.01 and *** *p* < 0.001 indicate significant differences from the Mock and siNC groups, respectively, as assessed by one-way ANOVA with Tukey–Kramer multiple comparisons tests. (**C**) Western blot analyses showing the protein levels of cyclin D1, CDK4, cyclin E1, and CDK2 in CCSCs after treatment with siLY6K or siNC for 48 h. Values are the mean ± SE (n = 3). *** *p* < 0.001 or **** *p* < 0.0001 indicates significant differences from the Mock and siNC groups as assessed by one-way ANOVA with Tukey–Kramer multiple comparisons tests. (**D**) Flow cytometric analyses of Annexin V-FITC/PI staining of CCSCs following siLY6K or siNC transfection for 48 h. Values are the mean ± SE (n = 3). Lower-left quadrant: viable cells; upper-right and lower-right quadrants: apoptotic cells; upper-left quadrants: necrotic cells. (**E**) Western blot analyses showing the protein levels of caspase-3 and cleaved caspase-3 in CCSCs after treatment with siLY6K or siNC for 48 h. Values are the mean ± SE (n = 3). LY6K, lymphocyte antigen 6, locus K; CCSC, colon cancer stem cells; CDK, cyclin-dependent kinase; PI, propidium iodide; FITC, fluorescein isothiocyanate; n.s., not significant.

MTS assays revealed that LY6K silencing inhibited CCSC proliferation compared to negative controls (Figure 4A). Flow cytometry analysis showed that LY6K silencing induced G1 phase arrest in CCSCs (Figure 4B). Mock, siNC, and siLY6K groups had 70.51% ± 2.41%, 74.12 ± 6.56%, 82.02 ± 3.12% of cells, respectively, in the G1 phase. Western blot analysis further showed reduced expression of CDK2 and CDK4 proteins, while cyclin D1 and cyclin E1 protein levels remained unchanged in siLY6K-transfected CCSCs compared to controls (Figure 4C). Annexin V-FITC/PI apoptosis assays showed no significant differences in apoptosis between siLY6K-transfected and control CCSCs (Figure 4D). Similarly, Western blot analysis showed no significant changes in caspase-3 and cleaved caspase-3 protein levels (Figure 4E). These results suggest that LY6K silencing inhibits CCSC proliferation by inducing cell cycle arrest rather than apoptosis.

### 3.5. LY6K-Silencing Suppresses the Growth of CCSCs in Xenografts and Promoted Mice Survival

**Figure 5:**
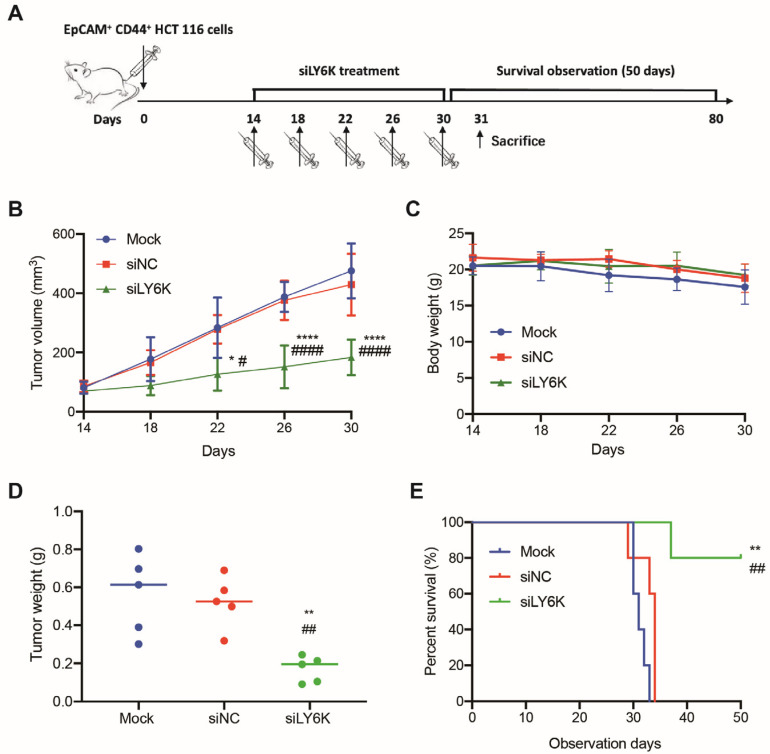
LY6K knockdown suppressed xenograft tumor growth and prolonged mice survival. (**A**) Scheme of an established murine colon cancer model of colon carcinoma. Average tumor volume (**B**), average body weight (**C**), and average tumor weight (**D**) (n = 5 mice per group). * *p* < 0.05, ** *p* < 0.01 or **** *p* < 0.0001 and ^#^ *p* < 0.05, ^##^ *p* < 0.01, or ^####^ *p* < 0.0001 indicates significant differences from the Mock and siNC group, respectively, as assessed by one-way ANOVA with Tukey–Kramer multiple comparisons tests. (**E**) Percentage of xenograft mice survival (n = 5 mice per group). ** *p* < 0.01 and ^##^ *p* < 0.01 indicates significant differences from the Mock and siNC group, respectively, as assessed by the Kaplan–Meier method and log-rank (Mantel–Cox) test.

A xenograft mouse model derived from CCSCs was used to evaluate the therapeutic potential of LY6K silencing. CCSCs were engrafted into BALB/c nude mice and allowed to grow until measurable tumors developed in all mice. siLY6K was then injected into peritumoral regions twice a week for a total of three weeks (Figure 5A). Regular measurements of tumor volume showed that LY6K silencing significantly decreased the tumor growth rate compared to the scrambled control (*p* < 0.0001, Figure 5B). The body weight of the mice did not change significantly among the groups (Figure 5C). On Day 31, the mice were sacrificed, and the tumor weights were measured, showing decreased tumor weights in the siLY6K group compared to the control groups (*p* < 0.01, Figure 5D). In the 50-day survival test, the siLY6K group showed a significantly higher survival rate compared to the control groups (*p* < 0.01, Figure 5E). By Day 50, 80% of the mice in the siLY6K group survived, while no mice in the control groups survived. These results indicate that LY6K-silencing improved the mice survival rate and suggest that LY6K may be a novel therapeutic target for colon cancer.

## 4. Discussion

CSCs are the main triggering factor of tumor progression, recurrence, and metastasis, and they are considered the major reason for therapy resistance and poor patient survival [23]. Despite the challenges, the continued investigation and development of new therapeutic agents targeting CSCs remain critical in order to prevent tumor relapse and metastasis after traditional anti-tumor therapies.

LY6/uPAR superfamily members, such as LY6E, LY6H, LY6K, and PSCA, are overexpressed in various cancers and contribute to tumor growth, invasion, and metastasis [24,25,26,27]. In this study, RT-qPCR analysis revealed that LY6K expression was elevated in CCSCs compared to HCT116 cells. LY6K overexpression has been linked to tumor progression, metastasis, and poor prognosis, thus making it a potential diagnostic or prognostic biomarker. Our study also showed that LY6K influences the biological behavior of CCSCs, including cell proliferation, cell cycle, migration, and invasion. Silencing of LY6K reduced the proliferation of CCSCs, which is consistent with previous studies, demonstrating that LY6K downregulation inhibits proliferation through the miR-500a-3p signaling axis in non-small cell lung cancer [28]. Flow cytometric analysis and Western blotting revealed that LY6K silencing induced G1 phase arrest by reducing CDK2 and CDK4 protein levels, without significantly affecting apoptosis. These results indicate that the decrease in CCSC proliferation is primarily due to cell cycle arrest. However, a study of ZNF252P AS1 in ovarian cancer showed that miR-324-3P overexpression promotes the apoptosis of ovarian cancer cells by negatively regulating LY6K [29]. Additional cell death mechanisms, such as necroptosis and autophagy, may be explored in future studies. Studies have shown that LY6K has been implicated in regulating key oncogenic pathways, particularly the TGF-β and ERK/MAPK signaling pathways [12,13]. LY6K may modulate the transition of TGF-β signaling from a suppressive to a promotive role, enhancing tumor progression and altering the cell cycle [11,30]. Additionally, LY6K may amplify signals from EGFR and activate the MAPK cascade. The ERK/MAPK signaling pathway is known to regulate CDKs and cyclins, which are key players in cell progression. The LY6K-driven activation of this pathway might directly influence G1 phase arrest by altering CDK activity and cyclin expression [12].

The ability of cell invasion and migration is critical in the process of tumor development. LY6K-silencing also significantly inhibited CCSC migration and invasion. These findings are consistent with studies showing that LY6K promotes migration, invasion, and immune escape in breast cancer cells by activating the Ras/ERK and TGF-β signaling pathway [13,14]. In our xenograft mouse model, LY6K silencing suppressed tumor growth and improved mice survival, supporting its therapeutic potential in colorectal cancer. 

Although the precise mechanisms underlying LY6K’s oncogenic effects in colorectal cancer remain unclear, previous studies suggest that LY6K overexpression may suppress T-cell development, thereby weakening the immune response against tumors [31]. Meanwhile, LY6K has been shown to be critical for cancer cells’ ability to evade immune surveillance [13]. A study revealed that the increased expression of Ly6K/E promotes cytokine-induced immune checkpoint molecules PDL1 and CTLA4 expression, enhances tumor-infiltrating T regulatory cells, and reduces natural killer cell activation. Additionally, the same study found that LY6K promotes gene signatures associated with drug resistance in breast cancer, emphasizing its role in immune evasion and therapy resistance. 

Our earlier studies on the LY6/uPAR superfamily member LY6D has shown that the silencing of LY6D expression suppressed colon cancer in xenograft mice [16]. Moreover, the results indicated that LY6D regulates CCSC proliferation and invasion in colon cancer through the MAPK pathway. pBRAF and pERK1/2, cascade kinases of the MAPK pathway, were downregulated after LY6D knockdown. CSCs are considered the source of tumor chemoresistance and contribute to tumor metastasis. A study showed that LY6D could serve as a biomarker for chemoresistance in CSCs [32]. LY6D was highly expressed in laryngeal cancer samples and was correlated with pathological T and clinical stages with cervical lymph node metastasis. LY6D facilities chemoresistance in laryngeal squamous cell carcinoma through mirR-509/β-catenin signaling pathway [33]. Further studies are needed to investigate the signaling pathways involved in LY6K-mediated oncogenesis to better understand its role and therapeutic potential in colorectal cancer. 

LY6K has also been explored as a target for cancer immunotherapies such as vaccines and T-cell-based therapies [34]. A LY6K-derived polypeptide vaccine has shown promising results in clinical trials, demonstrating the ability to induce effective immune responses, suppress tumor growth, and improve prognosis in patients with advanced solid tumors. A trial using a polypeptide vaccine targeting LY6K, DEPDC1, KIF20A, and FOXM1 in patients with high-grade glioma showed that the vaccine was well tolerated, elicited an effective immune response, and prevented disease progression for at least 6 months [35]. LY6K has been shown to stimulate cytotoxic T lymphocytes that recognized and killed esophageal and lung cancer cells that express these proteins [36]. A phase I trial for advanced esophageal squamous cell carcinoma in patients with HLA-A*2402 demonstrated that a combination of LY6K and TTK with CpG-7909 elicited potent LY6K-specific T cell responses. This vaccine combination induced antigen-specific CD8^+^ T cell responses and enhanced the innated immunity, showing promising immune activation for potential therapeutic application [37]. 

For siRNA drug development, it is important to consider potential off-target effects, which may result in unintended gene silencing, immunogenicity, or repressing translation of unintended targets [38,39]. siRNA target specificity, cross-species targeting, or even on-tissue target selectivity should be considered for siRNA design. Combination therapy, for example, combining LY6K silencing with immune checkpoint or pathway-specific inhibitors, advanced chemical modifications to enhance siRNA stability and reduce immunogenicity, and optimized delivery systems may minimize the off-target effects and thus improve therapeutic outcomes. Moreover, a comprehensive profiling ensures safety and specificity in clinical applications [40]. While LY6K is a promising target, challenges remain in translating these findings into clinical practice. These include optimizing delivery methods for LY6K-targetd therapies, ensuring immune responses are specific and durable, and avoiding immune evasion by tumor cells. Large-scale clinical trials are also necessary to validate LY6K as a safe and effective target across different cancer types.

In conclusion, LY6K expression is elevated in CCSCs, and its silencing inhibits CCSC proliferation by inducing G1 phase cell cycle arrest and suppressing migration and invasion. LY6K silencing significantly reduced tumor growth and improved survival in a colorectal cancer xenograft model. These findings suggest that targeting LY6K may be a promising therapeutic strategy to reduce tumor metastasis and therapy resistance in colorectal cancer.

## Figures and Tables

**Table 1:** The primer sequences used for the quantitative real-time PCR.

Target	Sequence (5′ → 3′)	Size (bp)
*GAPDH*	F: CAGGAGGCATTGCTGATGAT R: GAAGGCTGGGGCTCATTT	138
*LY6E*	F: CCGACCAGGACAACTACTGC R: ACACCAACATTGACGCCTTC	130
*LY6H*	F: ACAAGATGTGTGCCTCCTCC R: CACAAATCCTTCTCGCAGCA	121
*LY6K*	F: CTGACTGCGAGACAACGAGAT R: ATTTGCACCTCCTTGGGTTCT	124
*PSCA*	F: CAAAGCCCAGGTGAGCAACG R: CTGTGAGTCATCCACGCAGTT	148
*NANOG*	F: CTCCAACATCCTGAACCTCAGC R: CGTCACACCATTGCTATTCTTCG	115
*SOX2*	F: TGGACAGTTACGCGCACAT R: CGAGTAGGACATGCTGTAGGT	215
*C-MYC*	F: CACCAGCAGCGACTCTGAGGAG R: ACTTGACCCTCTTGGCAGCAGG	239
*OCT4*	F: GCAGCTTGGGCTCGAGAAGGAT R: AGCCCAGAGTGGTGACGGAGAC	269

F: forward; R: reverse.

## Data Availability

All datasets used and/or analyzed during this study are available from the corresponding author upon reasonable request.
